# Integrating linear optimization with structural modeling to increase HIV neutralization breadth

**DOI:** 10.1371/journal.pcbi.1005999

**Published:** 2018-02-16

**Authors:** Alexander M. Sevy, Swetasudha Panda, James E. Crowe, Jens Meiler, Yevgeniy Vorobeychik

**Affiliations:** 1 Center for Structural Biology, Vanderbilt University, Nashville, TN, United States of America; 2 Electrical Engineering and Computer Science, Vanderbilt University, Nashville, TN, United States of America; 3 Vanderbilt Vaccine Center, Vanderbilt University Medical Center, Nashville, TN, United States of America; 4 Department of Chemistry, Vanderbilt University, Nashville, TN, United States of America; The Pennsylvania State University, UNITED STATES

## Abstract

Computational protein design has been successful in modeling fixed backbone proteins in a single conformation. However, when modeling large ensembles of flexible proteins, current methods in protein design have been insufficient. Large barriers in the energy landscape are difficult to traverse while redesigning a protein sequence, and as a result current design methods only sample a fraction of available sequence space. We propose a new computational approach that combines traditional structure-based modeling using the Rosetta software suite with machine learning and integer linear programming to overcome limitations in the Rosetta sampling methods. We demonstrate the effectiveness of this method, which we call BROAD, by benchmarking the performance on increasing predicted breadth of anti-HIV antibodies. We use this novel method to increase predicted breadth of naturally-occurring antibody VRC23 against a panel of 180 divergent HIV viral strains and achieve 100% predicted binding against the panel. In addition, we compare the performance of this method to state-of-the-art multistate design in Rosetta and show that we can outperform the existing method significantly. We further demonstrate that sequences recovered by this method recover known binding motifs of broadly neutralizing anti-HIV antibodies. Finally, our approach is general and can be extended easily to other protein systems. Although our modeled antibodies were not tested *in vitro*, we predict that these variants would have greatly increased breadth compared to the wild-type antibody.

## Author summary

In this article, we report a new approach for protein design, which combines traditional structural modeling with machine learning and integer programming. Using this method, we are able to design antibodies that are predicted to bind large panels of antigenically diverse HIV variants. The combination of methods from these fields allows us to surpass protein design limitations that have been seen up to this point. We predict that if we tested these modified antibodies against HIV variants they would have greater neutralization breadth than any antibodies seen to this point.

## Introduction

Computational design has been used successfully by protein engineers for many years to alter the physicochemical properties of proteins [1,2]. In the simplest case, protein design involves optimizing the amino acid sequence of a protein to accommodate a desired 3-D conformation. This approach has been extended to related tasks such as protein-protein interface design, de novo design of protein binding molecules, design of self-assembling protein nano-cages, etc. [3–6]. Each of these examples involves the straightforward application of design methodologies to a single, static protein conformation. However, there is a need to extend protein design to apply to several conformations simultaneously. These approaches, referred to as multistate design (MSD), can be used to modulate protein specificity, model protein flexibility, and engineer proteins to undergo conformational changes [7–13]. Several methods have been developed to enable computationally expensive multistate design [14,15]. However, these methods all suffer from large energetic barriers that limit sampling in sequence space, resulting in sub-optimal designs [14]. In addition, these methods are severely limited in scale by the size and number of states that can be included. To address these limitations, we have developed a method that integrates structural modeling with integer linear programming to enable a fast global search through large ensembles of target states.

## Results

### Experimental workflow

**Fig 1:**
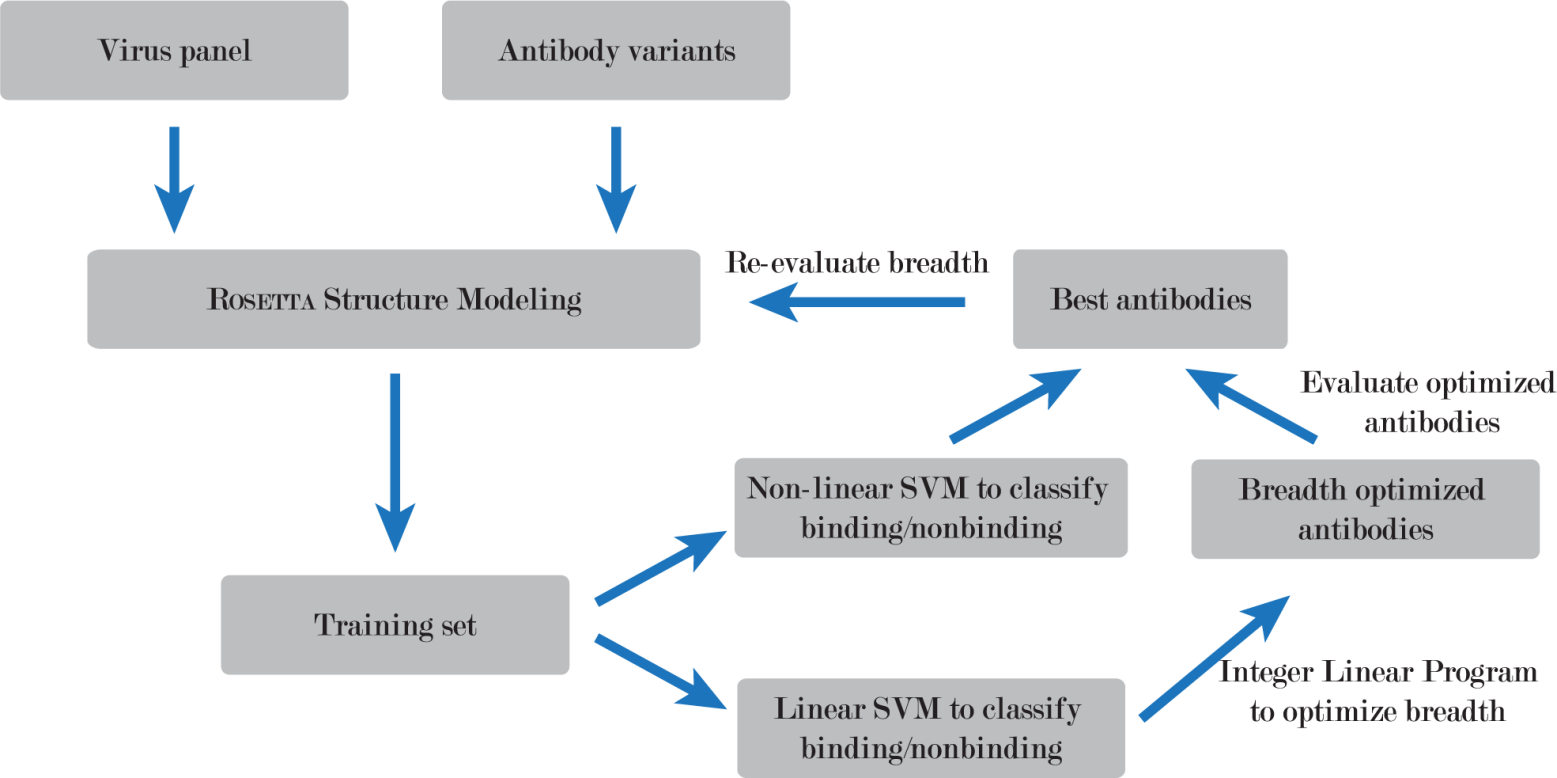
Experimental workflow of the BROAD design method. The method uses Rosetta structural modeling to generate a large set of mutated antibodies, support vector machines (SVM) to predict Rosetta energy from amino acid sequence, and integer linear programming to optimize breadth of binding across a set of viral proteins.

Our design algorithm, which we call BROAD (BReadth Optimization for Antibody Design) incorporates Rosetta-based structural modeling with integer linear programming to more easily traverse boundaries in the energy function (Fig 1). The experimental workflow involves generating a large training set of randomly mutated proteins, fitting a linear model (described below) to predict binding, and using integer linear programming to find an optimal antibody sequence balancing stability and binding with respect to a collection of target virus epitopes.

We applied this method to the problem of designing broadly binding anti-HIV antibodies. We modeled anti-HIV antibody VRC23 [16] against a set of 180 diverse viral proteins, creating antibody variants that were mutated randomly in the paratope region. The viral panel used was derived from Chuang G-Y, et al [17]. Based on known binding patterns of VRC23 we calculated the predicted binding energy that corresponds to observable binding, and searched antibody space using integer linear programming to optimize stability of the unbound antibody while achieving predicted 100% binding breadth to the 180 target viral proteins. We then used a non-linear Support Vector Machine classifier, trained on the entire dataset produced by Rosetta, to identify top sequences. Finally, we entered the top scoring sequences back into Rosetta structural modeling to measure the predicted breadth of antibody variants.

### Sequence-based linear classification and regression models to predict binding and stability

Our end goal is to design broadly binding and stable antibodies by searching the sequence space, i.e., to optimize the amino acids at each binding position of the antibody. The key challenge for this approach is that an exhaustive search in the combinatorial sequence space is intractable. To address this issue, we first propose to learn sequence-based linear classification and regression models to predict binding and stability from data. Building on these models, we formulate an integer program to accomplish global search in the antibody sequence space.

To generate our training set, we determined three contiguous stretches on the antibody that are in contact with the viral protein. These positions were determined to be residues 46–62, spanning FR2-CDR2-FR3; residues 71–74 in FR3; and residues 98-100b in CDR3 (S1 Fig). We then created randomly mutated antibody variants, modeled their binding poses using Rosetta, and used this data to train a binding classifier to predict Rosetta score and binding energy from amino acid composition.

The binding classifier is based on the assumption that the amino acids at the binding positions of the antibody interact with those on the binding positions of the virus. In particular, this model assumes that binding between an antibody and a viral protein is determined by two factors: a) the individual amino acids in each binding position of the antibody and the virus respectively and b) the effects of the pairwise amino acid interactions between the antibody and the virus respectively. To capture these, we construct a sequence-based binary feature vector from the input antibody and virus pair, which explicitly represents the individual and pairwise amino acid contributions. Let the input antibody-virus pair represented as vectors of amino acids, be denoted by (**a**, **v**). Let *b*(**a**, **v**) denote the Rosetta predicted binding energy for (**a**, **v**) and let Φ(**a**, **v**) denote the binary binding decision. We chose a threshold *θ* such that Φ(**a**, **v**) = +1 if *b*(**a**, **v**) ≤ *θ* (i.e., **a and v** bind) and Φ(**a**, **v**) = −1 otherwise. For evaluation of our approach, we choose the value of θ based on experimental neutralization data. This data is available as the experimental neutralization IC50 (in units of μg/ml) of VRC23 with the 180 virus sequences in the panel [17]. Lower values represent better neutralization potency and values that have ‘>50’ concentration represent a virus that is not neutralized by VRC23. Accordingly, VRC23 has a neutralization breadth of 63.5% on this panel. We set θ = -28.5 such that the VRC23 breadth of binding computed on the Rosetta generated data (sequences and the corresponding Rosetta binding scores) is consistent with the above experimental neutralization data.

**Fig 2:**
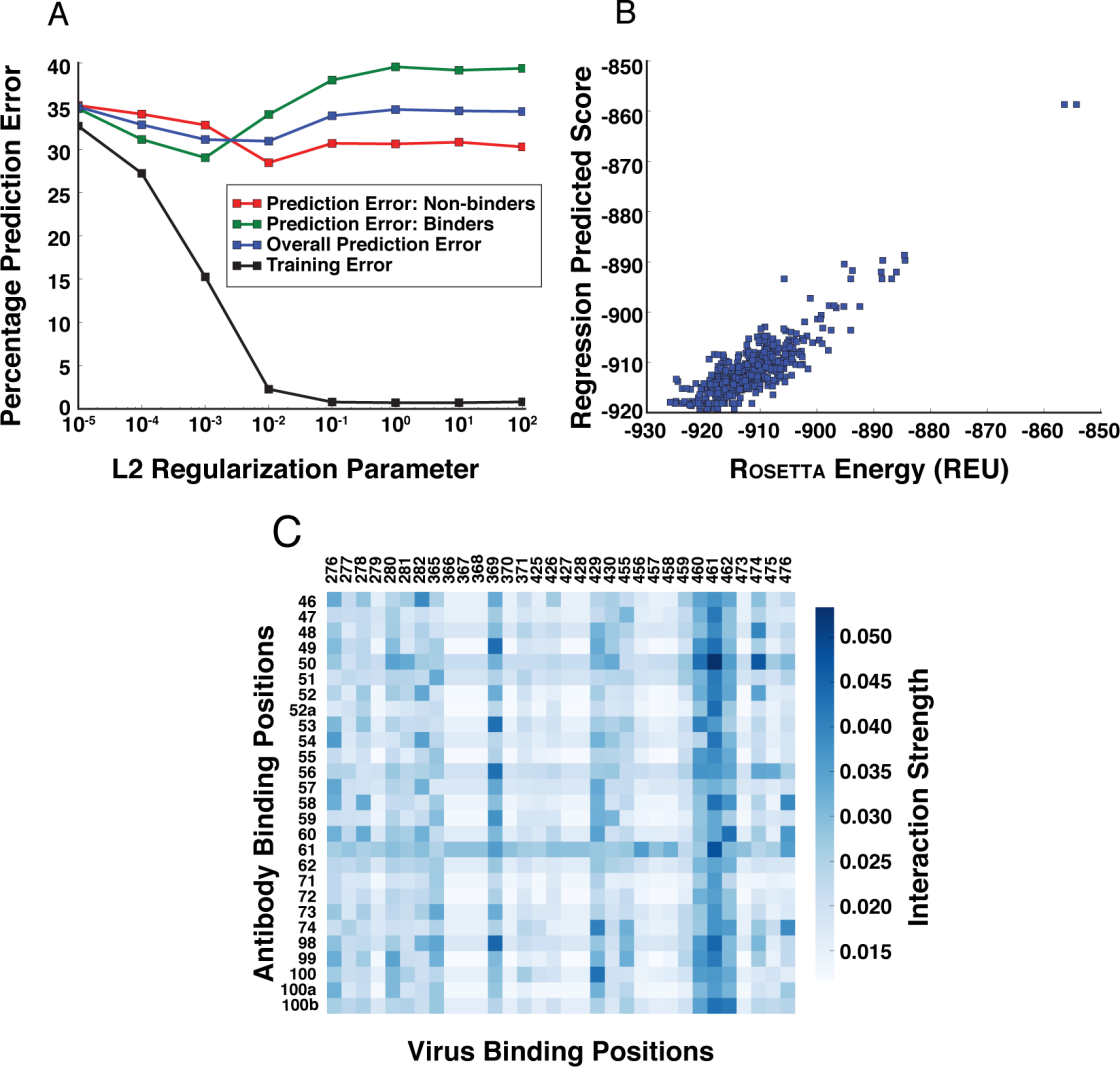
Training results for the linear classification: (a) 10-fold cross validation results. (b) Correlation between predicted score and Rosetta energy score in linear regression. (c) Interaction strength of each pairwise interaction between antibody and virus binding positions.

We learn the classifier Φ(**a**, **v**) as a linear Support Vector Machine (SVM) [18] using the binary feature set comprised of actual antibody and virus sequences along the corresponding binding sites, as well as all pairwise interactions of antibody and virus amino acids. The SVM classifier uses the Rosetta binding energy as the ground truth, and allows more efficient sampling by approximating the Rosetta score function by sequence alone. To optimize the L2 regularization parameter of the SVM, we performed 10-fold cross-validation on the full dataset, using 80% of the data for training and 20% for testing. Smaller parameter values enforce higher regularization and higher values lead to overfitting. The average prediction accuracy is shown in Fig 2A for different values of the L2 regularization parameter. We also plot the prediction error on the two classes: binders (+1) and non-binders (-1). The prediction accuracy is 67% on the test set using the optimized parameter (a random predictor would be at 50%). We observe that even if the prediction accuracy is relatively low, it provides reasonable signal within the subsequent breadth optimization step (discussed in the results section). Since the final decision is determined by solving the breadth optimizing integer linear program, our approach does not rely on a highly accurate classification model. In previous research [19], a similar model was introduced to predict Δ*G* values for interaction between PDZ domains and peptide ligands. The result was a 0.69 correlation coefficient in 10-fold cross validation. This model can also be interpreted to identify the important binding position pairs that contribute significantly to the final prediction. We plot this interaction strength for each pairwise interaction in Fig 2C (please refer to the methods section for details).

Next, we learned a linear regression model to predict the thermodynamic stability, using only the antibody amino acids as features. The prediction of thermodynamic stability is necessary to ensure that our designed antibodies can be expressed stably. To simplify the approach, we predicted the stability of the antibody-virus complex as a function of the antibody sequence only (note that we do not make this assumption during evaluation). Specifically, we constructed a binary feature vector restricted to amino acids in the antibody binding positions. Let *s*(**a**, **v**) denote the Rosetta stability for the pair (**a**, **v**). We learn a linear model Ψ(**a**) to predict *s*(**a**, **v**) for an antibody **a** (i.e., independent of the virus). To measure the accuracy of prediction, we computed the correlation coefficient between the true scores and the predicted scores. Interestingly, our assumption that stability scores are only weakly dependent on the virus protein sequence is borne out: we found a correlation of 0.85 between the predicted and actual stability energy score on the test set (Fig 2B).

### Algorithm

Given the classification and regression model learned from data, we formulate an integer linear program (ILP) to optimize the amino acids in the antibody sequence space to achieve both breadth and stability. The variables are the amino acids in the antibody binding positions. The objective function optimizes the predicted stability score (i.e., minimizes Ψ(**a**)). The constraints represent the condition that the designed antibody should bind to all the viruses in the panel, using binding predictions from Φ(**a**, **v**). We found that this problem was always feasible: there always existed some antibody sequence that could bind to all viral proteins based on our learned binding model. More generally, we can impose a minimal binding breadth criterion. This algorithm is outlined in S2 Fig.

Armed with these tools, we used the following protocol to generate a collection of candidate antibodies to be evaluated using Rosetta. First, we took a random subsample of the full training data corresponding to 100 out of the 180 virus sequences. Using only this subsample, we trained the binding and stability models, Φ(**a**, **v**) and Ψ(**a**) respectively. We then solved the ILP described above to compute a stable, broadly-binding antibody sequence, considering only the 100 out of 180 selected virus sequences (that is, we only constrain the ILP to bind to these 100 virus proteins, rather than the full set of 180). We repeated this procedure 50 times, to obtain 50 candidate antibody sequences. To validate these optimized antibody candidates, we predicted binding and stability scores using a model trained on all the data. In case of stability prediction, we used a linear model as described above (since the model is reasonably accurate). For binding prediction however, we trained a non-linear (radial basis function kernel) SVM for improved prediction accuracy. Each of the 50 candidate antibodies were scored using these models trained on all data, in terms of predicted binding breadth and stability, and 10 best candidates were then chosen for Rosetta evaluation using the full panel of 180 virus proteins. This procedure is outlined in S3 Fig.

### Redesign of VRC23 improves predicted breadth

**Fig 3:**
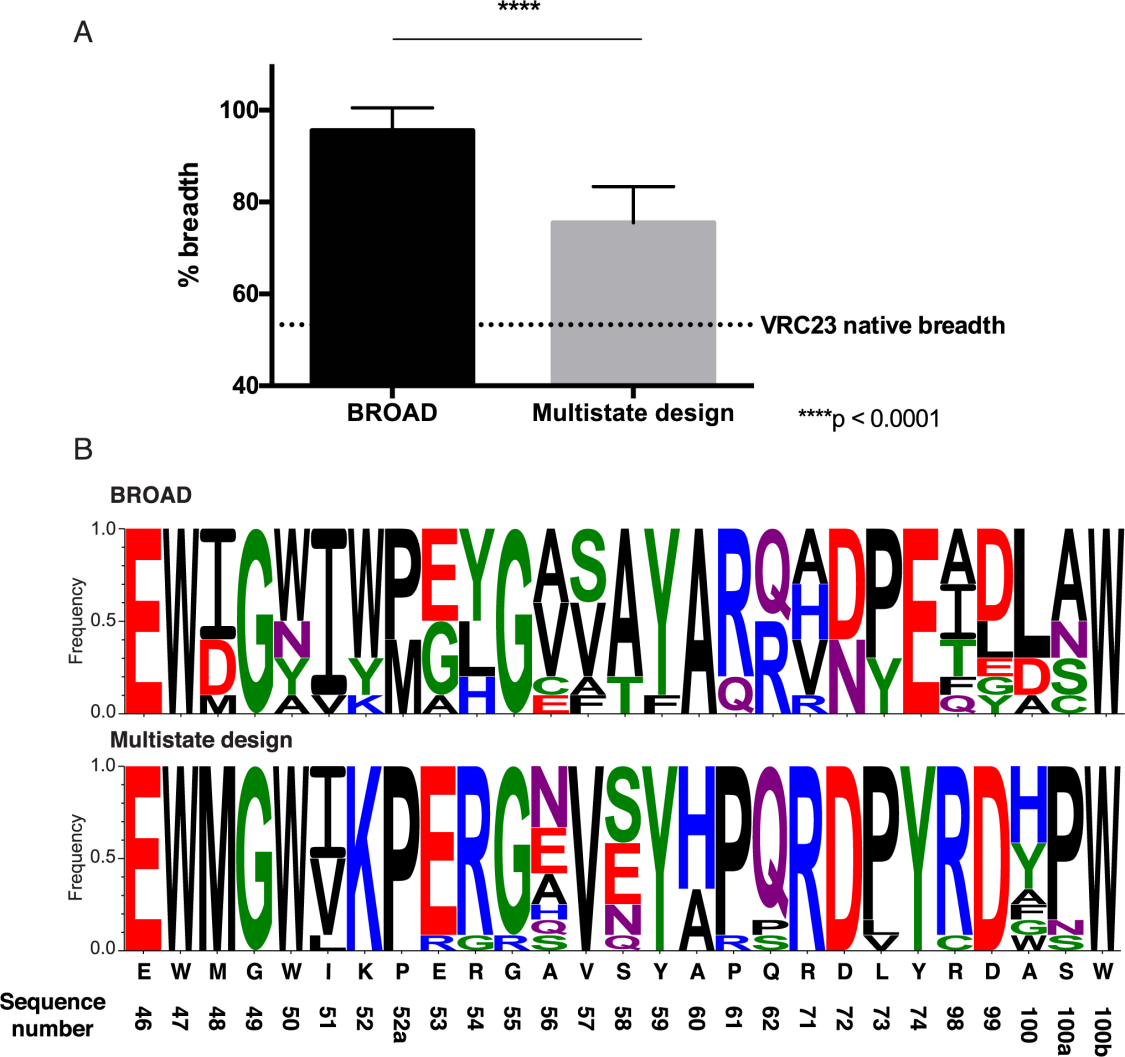
Redesign of VRC23 using integer linear programming increases predicted breadth over HIV viral strains. A. Predicted breadth of 10 redesigned antibodies generated either by BROAD or multistate design. Bars show mean and standard deviation of 10 sequences. Dotted line shows the predicted breadth of the native VRC23 antibody. B. Sequence logos of designed antibodies generated by BROAD or multistate design. Amino acids are colored based on chemical properties. The native VRC23 sequence is shown below.

**Fig 4:**
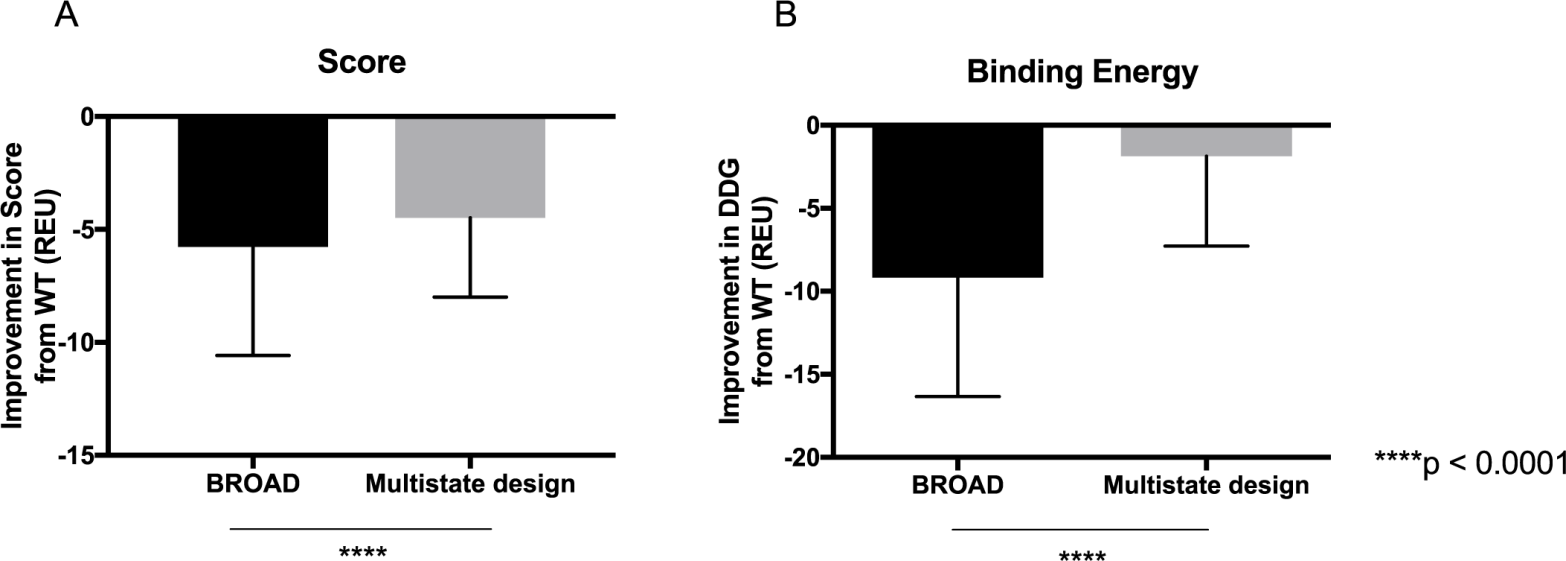
Score comparison of redesigned antibodies. The Rosetta score (A) and binding energy (DDG) (B) are shown for ten redesigned antibodies made either by BROAD or multistate design, paired with 180 viruses. Bar plots shown mean and standard deviation. Shown on the Y axis is difference between score/DDG between the redesigned antibody and wild-type.

After generating redesigned antibody sequences with predicted increases in breadth, we threaded these sequences onto the VRC23-gp120 complexes and subjected them to structural modeling to measure the change in predicted breadth. We refined the complexes using the Rosetta relax protocol–to test the accuracy of the Rosetta relaxed models, we compared the relaxed models to solved structures of gp120 viral variants and computed the root mean squared deviation (RMSD) over Cα atoms on gp120. We observed that the relax protocol recapitulates the gp120 conformations with an average RMSD of 2.2 Å, whereas the pairwise RMSD between gp120 conformations, representing the intrinsic flexibility of these molecules, is 1.8 Å (S1 Table). Considering that we substituted only residues at the binding site of the gp120 variants, and not the entire gp120 sequence, we consider that the variant gp120 conformations are recapitulated with sufficient accuracy for this experiment. As a control, we generated sequences using structure-based multistate design with the RECON method [14]. The RECON method uses Rosetta design combined with coordination between differing states to generate an antibody sequence with increased affinity for all target states. Using RECON to redesign antibody-antigen complexes has been benchmarked and been shown to generate germline-like, broadly binding antibodies [14]. We compared the 10 sequences created by BROAD to 10 sequences generated by RECON multistate design to compare the change in breadth to alternate approaches. We found that the BROAD method resulted in a significant increase in predicted breadth over the RECON multistate design method (Fig 3A). The BROAD-designed antibodies were able to achieve predicted breadth ranging from 86.1–100% of viruses, whereas multistate designed antibodies reached a predicted breadth of 62.8–85.6% of viruses. Notably, both methods were able to increase predicted breadth from the starting value of 53.3% for wild-type VRC23. This finding suggests that the wild-type VRC23 sequence is sub-optimal for breadth, which is supported by the observation that other known broadly neutralizing antibodies bind in a similar mode to VRC23 but with breadths exceeding 85% [20–23]. In addition, we observed that the BROAD method samples sequence space that is not sampled in multistate design (Fig 3B). We hypothesize that the BROAD method is able to cross energetic barriers that restrict sampling in traditional structure-based design methods, and is thereby able to generate antibodies with greater predicted breadth and lower energy. To support this hypothesis we analyzed the difference in score and binding energy for antibodies designed by BROAD and multistate design over the panel of viral proteins (Fig 4). BROAD was consistently able to generate lower energy antibody-antigen complexes, with a marked decrease in binding energy. This finding supports the hypothesis that BROAD is able to search sequences that are unavailable to multistate design, and that these new sequences have favorable score and binding energy.

### Designed residues recapitulate known binding motifs

**Fig 5:**
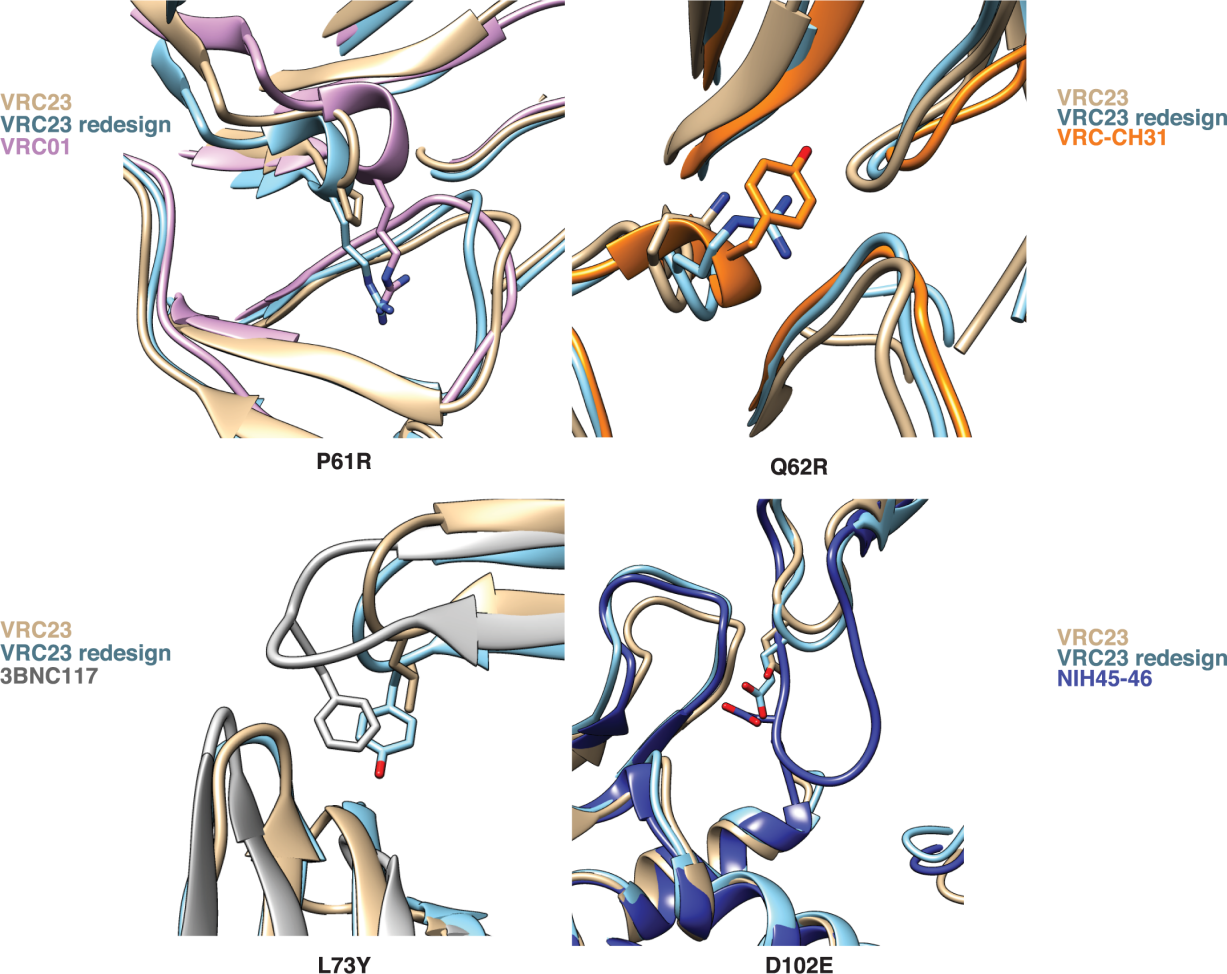
BROAD design recapitulates structural motifs of known broadly neutralizing antibodies. Residues that were mutated from the native VRC23 sequence were compared to known antibodies. Proteins shown are VRC23 (PDB ID: 4j6r); VRC01 (3ngb); VRC-CH31 (4lsp); 3BNC117 (4jpv); and NIH45-46 (3u7y).

A frequent problem in computational protein design is false positives–that is, sequences that are predicted to be favorable according to the score function, but are unable to recapitulate that activity *in vitro*. The Rosetta score function uses many approximations of energetic terms to enable faster simulations, and these approximations can introduce inaccuracies [24,25]. To reduce the possibility that the redesigned VRC23 variants are scored favorably due to inaccuracies in the score function, we compared the designed residues introduced by BROAD to structural motifs of known broadly neutralizing antibodies (Fig 5). In several cases, the residues introduced by BROAD mimicked a known interaction of an existing antibody. For example, position 61 was mutated from proline in VRC23 to arginine (Fig 5, top left). The broadly neutralizing antibody VRC01 has an arginine that occupies similar space to the designed arginine [20]. This phenomenon can be observed for several different broadly neutralizing antibodies, such as VRC-CH31, 3BNC117, and NIH45-46, all of which target the CD4 binding site, but at slightly different orientations [20–22,26]. We observed several examples of this type of recapitulation. Mutation Q62R on VRC23 placed an arginine residue to fill space that is occupied by a tyrosine on VRC-CH31 (Fig 5, top right)—this mutation fills a void at the interface to improve antibody-antigen packing. Mutation L73Y places an aromatic group overlapping with the position of a tyrosine in antibody 3BNC117, which also improves packing with the antigen (Fig 5, bottom left). Lastly, the D102E mutant on the CDRH3 places a carboxylic acid group in the same position as a glutamic acid on NIH45-46, improving electrostatic interactions with the antigen (Fig 5, bottom right). This observation is remarkable due to the fact that the antibody loops occupy different space, but redesigned residues are able to mimic the interactions of the broadly neutralizing antibody side chains. In addition, it is worthwhile to note that out of these four mutants that recapitulate known broad motifs, three were unobserved in the sequences sampled by multistate design (Fig 3B).

**Fig 6:**
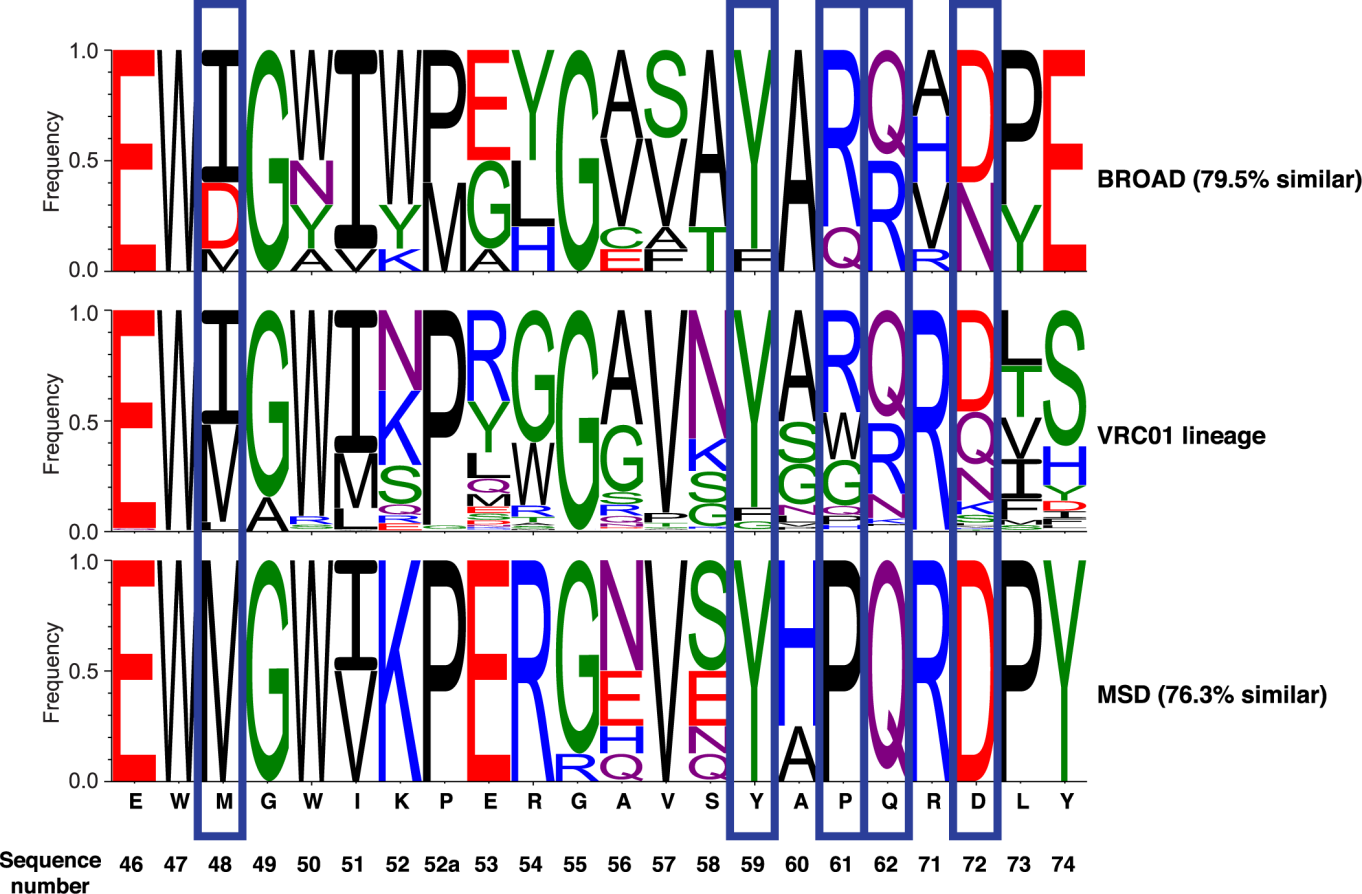
Sequences from BROAD design recapitulate sequences observed in the lineage of broadly neutralizing antibody VRC01. For BROAD and MSD sequences a percentage similarity to the VRC01 lineage was computed (similarity shown in parenthesis). Blue boxes highlight positions where BROAD samples an amino acid that is present in the VRC01 lineage but was not sampled by MSD. The VRC23 native sequence is shown below.

As an additional comparison, we identified 1,041 sibling sequences of known broadly neutralizing antibody VRC01, that were isolated in a previous study [27]. These siblings presumably represent the sequence space accessible to VRC01, and are a good test case to compare how well our design algorithms are capturing natural sequence variation in a broad HIV antibody. Since these sequences have CDRH3 loops of different lengths we were not able to include the portion of the binding site corresponding to the CDRH3 loop–however we compared the rest of the binding site to the sequences seen in the VRC01 lineage (Fig 6). We observe that at several positions, BROAD samples sequences that are present in the VRC01 lineage but absent from MSD-sampled sequences (Fig 6, blue boxes). For example, at the third position in the binding site isoleucine is sampled at a high frequency in BROAD and VRC01 lineage sequences, but is never sampled by MSD (Fig 6). We highlight a total of five positions where BROAD outperforms MSD in sampling sequences that are seen in the VRC01 lineage. To quantify the sequence similarity we computed a sum of squared difference between the two matrices and normalized the values to 100% [14,28]. According to this metric the sequences sampled by BROAD are 79.5% similar to those from the VRC01 lineage, whereas those sampled by MSD are only 76.3% similar. We conclude that BROAD more accurately recapitulates motifs known in broadly neutralizing antibodies.

## Discussion

### Summary of results

In this paper we describe the development of a new protein design method that we call BROAD. This method uses structural modeling with Rosetta combined with integer linear programming optimization techniques to rapidly search through sequence space for broadly binding antibodies. We validated this method by computationally optimizing the amino acid sequence of the broadly neutralizing anti-HIV antibody VRC23. After modeling VRC23 variants *in silico* we were able to generate VRC23 variants with a predicted breadth of 100% over the simulated viral panel, compared to a predicted 53% breadth for the wild type antibody. This outcome represents a substantial step forward in protein design, and our methodologies can be used to address a wide variety of protein design problems in which traditional structural models are insufficient.

Although we did not test antibody variants *in vitro* in this study, we predict that the computationally designed variants will have greater breadth against the HIV viral panel. However, we note several caveats with respect to experimental validation of these antibodies. Since this experiment was designed as a computational proof of principle, we modeled only the amino acids at the antibody binding interface of gp120, and not the entire gp120 sequence. This led to gp120 models with ~2 Å accuracy (S1 Table), which we consider sufficient for validating our design principles but not necessarily for experimental validation. Future directions in this work include optimizing protocols for gp120 homology modeling to reduce this discrepancy and enable experimental validation.

### Backbone optimization in protein design

A distinct advantage of the BROAD method is the ability to truly incorporate backbone movement into protein design. Many protein design methods have been developed that incorporate backbone ensembles to some degree [11,14,29,30]–however, this work typically involves either pre-generating large backbone ensembles, many of which may be redundant, or introducing backbone movement iteratively after steps of sequence design. In our approach, since we are relaxing the backbone of all mutants before fitting the sequence-based predictor, we were able to design sequences that may be slightly sub-optimal on the starting backbone coordinates, but can be highly favorable when a slight backbone relaxation is applied. This approach allows us to search sequence space that is not accessible to other methods, which are highly constrained to the initial backbone coordinates. We observed that the BROAD-generated sequences are not sampled by Rosetta design using the RECON method, and indeed are more favorable according to the Rosetta energy function. Therefore, we conclude that we are searching a “blind spot” in the sequence space that is missed by traditional design.

### Application to HIV immunology

This approach to research could be of great utility to the field of HIV immunology. A longstanding goal of the field is discovering broadly neutralizing antibodies as the basis of a rational structure-based vaccine strategy [31–33]. Much work has gone into redesigning existing antibodies to increase their breadth and potency [3,21]. However, HIV is known for its variability, and with this variability comes a difficulty in generating a single antibody with potent neutralization against all possible variants. The BROAD method addresses this problem by enabling rapid redesign of known antibodies against viral panels of arbitrary size. This technology can be used in the future as part of the antibody discovery and characterization process, by rapidly searching sequence space for variants for greater breadth. In addition, protein design also has been used on the reverse side of the vaccination problem, namely, to design a vaccine with high affinity for antibodies of interest [34–36]. We can foresee the application of the BROAD method to this problem as well, by optimizing immunogens for recognition of germline precursors of known broadly neutralizing antibodies.

## Materials and methods

### Structural modeling

The VRC23-gp120 complex used for modeling was from the Protein DataBank (PDB ID: 4j6r). The structure was downloaded from the PDB (www.rcsb.org) and processed manually to remove water and non-protein residues. The CH1 and CL1 domains of the antibody structure were removed from the structure manually, and the structure was renumbered starting from residue 1. To select binding sites on the antibody and virus, we applied a distance cutoff of 4 Å from the opposing protein chain, where any residue with a heavy atom within 4 Å of a heavy atom on the opposing protein was considered to be at the binding site. Distance calculations were done using PyMol visualization software [37]. We expanded this binding site to several neighboring residues to include contiguous stretches of at least four residues to constitute a binding site. A total of 27 residues on the antibody were included in the binding site. We similarly determined a viral binding site to use for structural modeling. This site included 5 contiguous stretches that were determined to be in contact with VRC23 (32 positions total). These positions were 276–282; 365–371; 425–430; 455–462; and 473–476 (HXB2 numbering). To model gp120 variants, we performed a multiple sequence alignment using ClustalW [38] of the variant sequences with the gp120 in the crystal structure (Q23.17), and substituted the corresponding amino acids at the binding site using Rosetta side chain optimization [24].

### Training set

To generate a training set of structural models, we made random antibody substitutions in the previously defined binding site. Each antibody variant had five randomly selected amino acid mutations. Viral variants were taken from a set of 180 known HIV gp120 sequences [17]. We chose random combinations of antibody variants and viruses, as well as the native antibody sequence with all 180 viruses, for a total of 2200 antibody-virus pairs to serve as the training set. All antibody-virus pairs were subjected to an energy minimization via the Rosetta relax protocol, which involves iterative rounds of side chain repacking and backbone minimization with an increasing repulsive force [39]. 50 models of each antibody-virus pair were generated by Rosetta relax, and the lowest scoring model was used for further evaluation. The talaris2013 score function was used for all Rosetta simulations.

### Linear classification and regression

Our data-driven sequence-based model to learn amino acid contributions to binding and stability is similar to the graphical model approach proposed in [19]. Let *N*_*a*_ and *N*_*v*_ denote the number of binding positions on the antibody and the virus respectively. Let $A=\{A_{1},A_{2}\ldots A_{N_{a}}\}$ be a set of discrete variables representing the amino acids in the binding positions of the antibody. Each *A*_*i*_ takes values in the set of *M* = 20 amino acids. Similarly, let $V=\{V_{1},V_{2}\ldots V_{N_{v}}\}$ represent the variables for the virus-binding positions. The inputs for binding prediction are the antibody sequence $a=\{a_{1},a_{2}\ldots a_{N_{a}}\}$ and virus sequence $v=\{v_{1},v_{2}\ldots v_{N_{v}}\}$ where *a*_*i*_ and *v*_*j*_ are the amino acid values for the variables *A*_*i*_ and *V*_*j*_. Amino acid contributions to binding can be modeled as a bipartite graph in which nodes for **A** and **V** represent the amino acids and the edges Ω ⊆ **A** × **V** represent the pairwise amino acid interactions. Each node *a*_*i*_ and *v*_*j*_ has associated weight vector **x**_*i*_ and $y_{j}\in R^{M}$. The edge (*i*, *j*) between nodes *a*_*i*_ and *v*_*j*_ has an associated weight matrix $Q_{ij}\in R^{M\times M}$ to represent the position specific contribution to binding for each amino acid pair, where $q_{kl}^{um}$ is the *um*th entry of matrix *Q*_*ij*_. Consequently, given **a** and **v**, the binding score varies as the sum of individual amino acids and pairwise interaction effects. Given this setting, **a** and **v** are predicted to bind, i.e., Φ(*a*, *v*) = +1 (*b*(*a*, *v*) ≤ *θ*), if

$$\sum_{i=1}^{N_{a}}\sum_{j=1}^{M}x_{ij}a_{ij}+\sum_{i=1}^{N_{v}}\sum_{j=1}^{M}y_{ij}v_{ij}+\sum_{k=1}^{N_{a}}\sum_{l=1}^{N_{v}}\sum_{u=1}^{M}\sum_{m=1}^{M}a_{ku}q_{kl}^{um}\;v_{lm}+c\leq 0$$ (1)

where *c* is the intercept term and *a*_*ij*_ and *v*_*ij*_ are binary indicator variables that take the value 1 if amino acid *j* is present at position *i* (∑_*j*_ *a*_*ij*_ = 1, ∑_*j*_ *v*_*ij*_ = 1 ∀ *i*). The $q_{kl}^{um}$ term represents *Q*_*kl*_(*u*, *m*). These weights can be learned efficiently using a linear support vector machine (SVM) classifier. The feature vector **f** consists of *N*_*a*_ × *M* binary antibody features, *N*_*v*_ × *M* binary virus features and *N*_*a*_ × *N*_*v*_ × *M* × *M* binary pairwise interaction features corresponding to **x**, **y** and *Q* respectively. Given a set of *d* training instance-label pairs (**f**_**i**_, *l*_*i*_), *i* = 1 … *d*, *l*_*i*_ = {+1, −1}, a L2-regularized linear SVM generates a weight vector **w** by solving the following unconstrained optimization: $\min_{w}\frac{1}{2}w^{T}w+\lambda \sum_{i=1}^{d}{\left(\max\left(1-l_{i}w^{T}f_{i},0\right)\right)}^{2}$, where *λ* > 0 is the L2 regularization parameter. Smaller *λ* values enforce higher regularization. The second term is the squared hinge loss function. The decision function is given by sign (**w**^*T*^**f**). We used the LIBLINEAR SVM implementation [40] to learn the classifier. Finally, the weights **x**, **y** and Q are retrieved from the combined weight vector **w**.

On each training set of the viruses, we trained this classifier and saved the weights and the intercepts for future use in optimization. In our example, *N*_*a*_ = 27 and *N*_*v*_ = 32. To tune the regularization parameter *λ* of SVM, we performed 10-fold cross-validation on the full dataset, using 80% of the data for training and 20% for testing. The average prediction accuracy is shown in Fig 2 for different values of the L2 regularization parameter *λ*. As expected, higher *λ* values lead to overfitting. We simultaneously plot the prediction error on the two classes: binders (+1) and non-binders (-1). We chose *λ* = 0.001 for our experiments based on the bias-variance trade-off (corresponding to 33% test error).

The above model can be interpreted to identify the important binding positions on the antibody and the virus side, i.e., the pairs that contribute significantly to the final prediction. Specifically, we denote the Euclidean norm of the coefficient matrix of interactions *Q*_*ij*_, for each position pair as the strength of interaction between those positions. We plot this interaction strength for each pairwise interaction in Fig 2C.

The linear regression model Ψ(**a**) predicts the stability scores as a function of the antibody sequence features:

$$\Psi \left(a\right)=\sum_{i=1}^{N_{a}}\sum_{j=1}^{M}x_{ij}^{s}a_{ij}+c^{s}$$ (2)

where $x^{s}\in R^{M}$ is the weight vector in regression and c^*s*^ is the intercept. Given a set of *d* training instance-score pairs (**a**_*i*_, *s*_*i*_) *i* = 1 … *d*, (*s*_*i*_ = *s*(**a**_*i*_, **v**_*i*_), so there are multiple scores for the same antibody feature vector), the regression objective with *l*_*1*_ (sparse) regularization is given by: $\min_{x^{s}}\frac{1}{2d}{\left(\|{\left(x^{s}\right)}^{T}a_{i}+c^{s}-s_{i}{\|}_{2}\right)}^{2}+\alpha \|x^{s}{\|}_{1}$, where the first term is the least squares penalty, *α* is the regularization parameter and ∥ **x**^*s*^ ∥_1_ is the *l*_*1*_-norm of the weight vector. We used the Lasso implementation in scikit-learn [41] to learn this model. To measure the effectiveness of the prediction, we computed the correlation coefficient between the Rosetta calculated stability scores (in Rosetta energy units, or REU) and the scores predicted by regression. We performed a 10-fold cross validation experiment similar to linear classification, with 80% of the data for training and 20% for testing. Based on this parameter tuning, we chose *α* = 0.01 with an average correlation of 0.85 between predicted and actual stability energy score. Again, for each training set of viruses, we learn this model and save the weights and the intercept for the optimization in the next step.

### Breadth maximization integer program

We leverage the weights in the binding and stability prediction models Φ(**a**, **v**) and Ψ(**a**) to formulate an ILP for optimization in the antibody sequence space. The objective is to minimize stability score. The constraints enforce the condition that the designed antibody should bind to each virus sequence in the training set. Finally, we add the constraint that the binary variables at each antibody binding position should sum to 1, i.e., each position admits one amino acid. The ILP is given by the following:

$$\begin{array}{l} \mathrm{minimize}\;\sum_{k=1}^{N_{a}}\sum_{u=1}^{M}(x_{ku}^{s})a_{ku}\\ subject\;to\\ \sum_{k=1}^{N_{a}}\sum_{u=1}^{M}(\sum_{l=1}^{N_{v}}\sum_{m=1}^{M}q_{um}^{kl}v_{lm}^{n}+x_{ku})\;a_{ku}+\sum_{i=1}^{N_{v}}\sum_{j=1}^{M}y_{ij}v_{ij}^{n}+c\leq -\epsilon ,\;\forall \;n\in 1,\ldots ,t\\ \sum_{u=1}^{M}a_{ku}=1,\;\forall k,\;a_{ku}\in \{0,1\} \end{array}$$

where *ϵ* = 0.0001 (which constrains that the antibody binds to all virus variants in the dataset, with a slight margin to ensure that binding is strictly below the 0 threshold). We used CPLEX version 12.51 to solve the above ILP. We solve this optimization problem for each binding and stability model learned for data obtained from randomly chosen 100 virus variants (from the dataset in which all 180 are represented).

### Non-linear classification for binding prediction

Our final step is to take 50 antibodies generated using the integer program above from 50 random subsets of data, and choose the top 10 candidates to evaluate with Rosetta. This decision is based on a non-linear model of binding learned on the full dataset which includes all 180 viral variants, combined with a full-dataset linear model of stability. The top 10 most stable antibodies from all which are predicted to have 100% binding breadth are then chosen for evaluation. The linear model of stability is identical to what we had described above.

For the non-linear model of binding we use a kernel support vector machine with the radial basis function (RBF) kernel. This model uses the same feature set as the linear model. The kernel function enables learning in a high-dimensional, *implicit* feature space without explicitly computing the coordinates of the data in that space. The RBF kernel of two feature vectors **f** and **f**′ is defined as:

$$K(f,f^{\prime })=\exp\left(-\frac{∥f-f^{\prime }{∥}^{2}}{2\sigma^{2}}\right),$$

where ∥ **f** – **f**′ ∥^2^ is the squared Euclidean distance between the two feature vectors, and *σ* is a free tunable parameter. Consequently, we have two free parameters to tune: the regularization parameter *λ*, and the RBF kernel parameter *σ*. Similar to the earlier set-up, we used 80% data for training and 20% for testing in a 10-fold cross validation experiment to tune these. We performed a grid-search over all pairwise combinations of *σ* and *λ* values in 10^−2^ to 10^2^. The LIBSVM implementation in scikit-learn was used to train the RBF SVM. We chose the model with *σ* = 0.01 and *λ* = 1 corresponding to the prediction accuracy of 68%.

All learning and ILP experiments were performed on a 2.4GHz hyper threaded 8-core Ubuntu Linux machine with 16 GB RAM.

### RECON multistate design

VRC23 was placed in complex with all 180 viruses and designed via RECON multistate design to increase predicted breadth across the panel. Models of viral variants were created as previously described, by substituting amino acids at the binding site. All VRC23-gp120 pairs were refined by Rosetta relax with constraints to the starting coordinates to prevent the backbone from making substantial movements. Constraints were placed on all Cα atoms with a standard deviation of 0.5 Å. All residues at the binding site of VRC23 were included in design, for a total of 27 residues. The RECON protocol was run in parallel over 180 processors (manuscript describing parallelization in preparation), with four rounds of design and a ramping convergence constraint [14]. The binding sites on both the antibody and gp120 chain was subjected to backrub movements between rounds of design to increase sequence diversity [42]. A total of 100 designs were generated. Sequences generated by both BROAD and RECON methods were visualized using the WebLogo tool [43].

### Sequence validation

To compare sequences generated by BROAD optimization and RECON multistate design, we threaded the optimized antibody sequences over the unprocessed VRC23-gp120 complexes, and subjected these complexes to Rosetta relax to determine the score and binding energy of optimized antibodies vs. wild-type. 50 models were generated for each complex, and the lowest scoring model was used for evaluation. To compare native and optimized VRC23 sequences, we compared the total energy of the VRC23-gp120 complex as well as the binding energy (DDG), defined below:

$$DDG=E_{complex}-\left(E_{Ab}+E_{Ag}\right)$$

where E_Ab_ and E_Ag_ are the energies of the antibody and antigen alone, respectively. Structures of modeled VRC23-gp120 complexes were visualized using Chimera software [44].

### Comparison to VRC01 lineage sequences

VRC01 lineage sequences were derived from a previous study [27]. The 1,041 curated heavy chain sequences we used in this analysis are available in GenBank with accession numbers KP840719–KP841751. To compare sequence profiles we used a modified Sandelin-Wasserman similarity score, as described in [14,28]. Briefly, this score was calculated by computing the sum of squared difference for each amino acid frequency at each position, which was then subtracted from two and normalized to yield a percent similarity for each position and summed over all designed positions to give an overall similarity score.

## Supporting information

S1 Fig Binding site of VRC23 shown in context of the antibody-antigen complex. The binding site encompasses FR2, CDR2, FR3 and CDR3 regions of the antibody heavy chain.
(TIF)

S2 Fig Pseudocode describing the Integer Linear Program. (TIF)

S3 Fig Pseudocode describing the BROAD algorithm for design of broadly binding antibodies. (TIF)

S1 Table Rosetta relaxed models used in BROAD optimization were compared to solved structures of gp120 viral variants and the root mean squared deviation (RMSD) was computed over Cα atoms on gp120. The relax protocol recapitulates the gp120 conformations with an average RMSD of 2.2 Å.
(DOCX)

S1 File Protocol capture to run all relevant protocols described in the manuscript. (DOCX)
