# Supplementary figures and images for: Integrating linear optimization with structural modeling to increase HIV neutralization breadth
Source: PLoS Comput Biol. 2018 Feb 16;14(2):e1005999. doi: 10.1371/journal.pcbi.1005999 (PMC5833279; doi:10.1371/journal.pcbi.1005999)

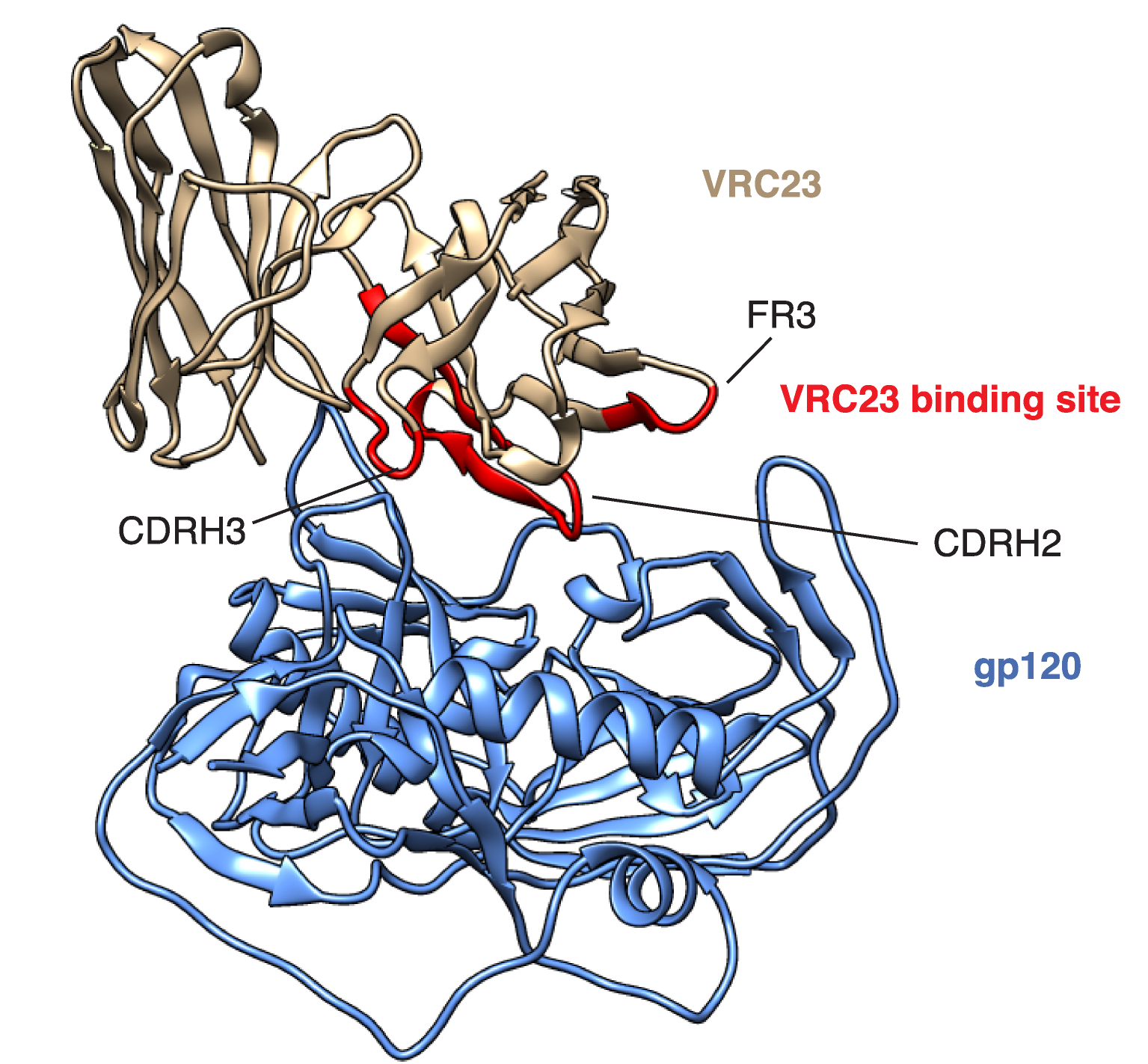

Supplement: S1 Fig — The binding site encompasses FR2, CDR2, FR3 and CDR3 regions of the antibody heavy chain. (TIF) [file pcbi.1005999.s001.tif]

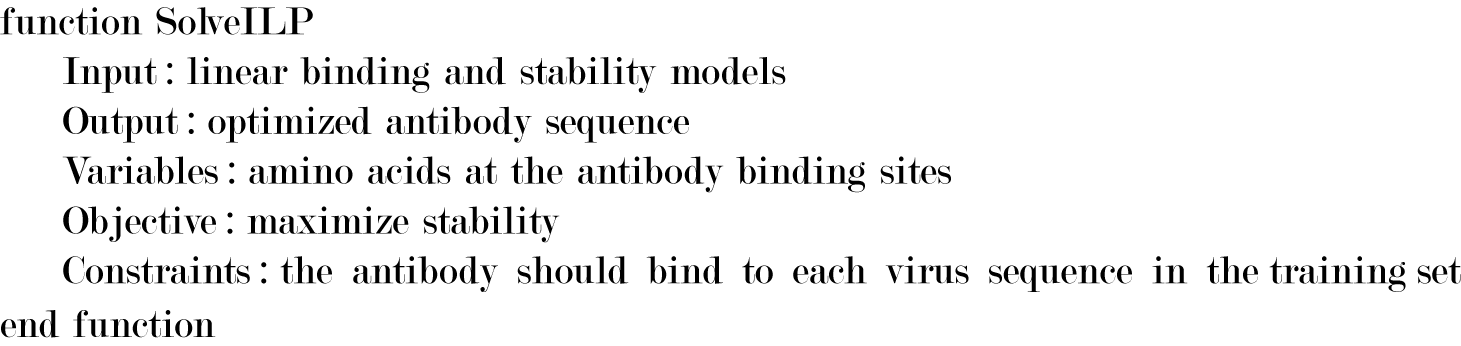

Supplement: S2 Fig — (TIF) [file pcbi.1005999.s002.tif]

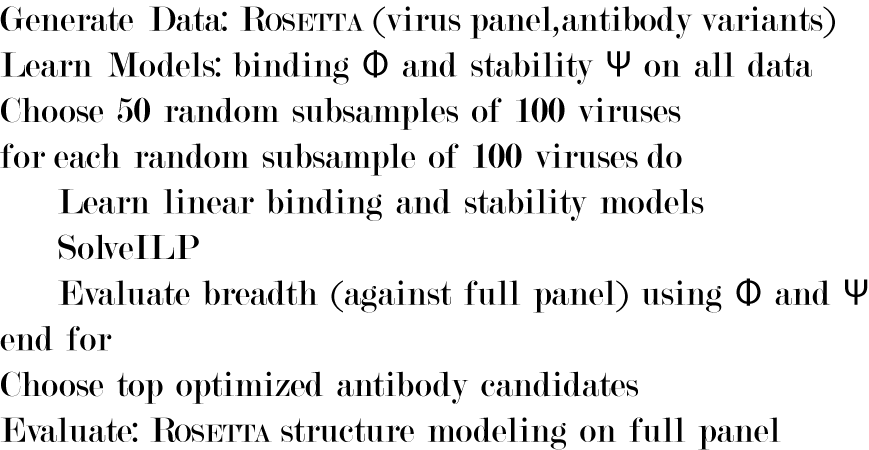

Supplement: S3 Fig — (TIF) [file pcbi.1005999.s003.tif]
