# Supplementary material for: Integrating linear optimization with structural modeling to increase HIV neutralization breadth
Source: PLoS Comput Biol. 2018 Feb 16;14(2):e1005999. doi: 10.1371/journal.pcbi.1005999 (PMC5833279; doi:10.1371/journal.pcbi.1005999)
Supplement: S1 Table — The relax protocol recapitulates the gp120 conformations with an average RMSD of 2.2 Å. (DOCX) [file pcbi.1005999.s004.docx]

| **gp120 for indicated HIV strain** | **PDB ID** | **RMSD between relaxed model and crystal structure** |
| --- | --- | --- |
| Q23-17 | 4j6r | 1.4 |
| YU2-DG | 3tgq | 2.1 |
| Du172-17 | 5te7 | 2.8 |
| RHPA-7 | 5t33 | 2.2 |
| X2088-c9 | 5te4 | 2.5 |
| ZM109-4 | 3tih | 1.9 |
| JRCSF-JB | 4r2g | 2.4 |
| HXB2-DG | 1g9m | 2.5 |
| Q842-d12 | 4xmp | 2.4 |
| **Average** |  | **2.2** |

Table S1: Rosetta relaxed models used in BROAD optimization were compared to solved structures of gp120 viral variants and the root mean squared deviation (RMSD) was computed over Cα atoms on gp120. The relax protocol recapitulates the gp120 conformations with an average RMSD of 2.2 Å.
