# Supplementary material for: Integrating linear optimization with structural modeling to increase HIV neutralization breadth
Source: PLoS Comput Biol. 2018 Feb 16;14(2):e1005999. doi: 10.1371/journal.pcbi.1005999 (PMC5833279; doi:10.1371/journal.pcbi.1005999)
Supplement: S1 File — (DOCX) [file pcbi.1005999.s005.docx]

**Protocol capture**

**Introduction**

The following is a protocol capture of how to run the BROAD multistate design method. It will go through an example of making models of an antibody-antigen complex of VRC23 in complex with HIV gp120, generating a training set of randomly mutated antibodies, fitting the structural models to a classifier, and using integer linear programming to search in sequence space for a broadly binding antibody.

All Rosetta commands for this publication were run with version 52d173bb0f823b30c009662efb2eb7e635176fc4, from Jan 2016. Note that all analysis scripts will only function properly if they are in the correct directory as provided.

All materials from this protocol capture can be downloaded from https://github.com/sevya/broad_protocol_capture

**Dependencies:**

Several scripts require the use of Python 2.7 as well as the Biopython package (<https://github.com/biopython/biopython.github.io/)>. The scikit learn package is also required to fit the classification model (<https://github.com/scikit-learn/scikit-learn>). Lastly IBM cplex is required for solving the integer linear program (<https://www.ibm.com/analytics/data-science/prescriptive-analytics/cplex-optimizer>). We recommend installing all necessary packages before beginning this protocol.

**PDB preparation**

First, PDB structure 4j6r was downloaded from the RCSB and manually processed in PyMol. All waters were removed from the structure and non-protein residues were also removed. The antibody was processed to remove the constant domain of the Fab fragment – this was done to reduce the total size of the system and simulation time. In this case, residues 114 – 214 on chain H and residues 108 – 214 on chain L were removed. The complex was then saved and renumbered using a python script. The following script both renumbers the PDB chains and reorders the chains, so that chains H and L come first in the file. Note that this does not change any of the atomic coordinates in the structure, only reorders them.

python reorder_pdb_chains.py --new_chain_order H,L,G 4j6r.pdb 4j6r_renum.pdb

To select binding sites for the antibody and antigen, we selected residues within a 4 Å cutoff of the opposing chain using PyMol. We used the following commands to do so:

select ab_binding_site, byres( chain H+L within 4 of chain G )

select ag_binding_site, byres( chain G within 4 of chain H+L )

After expanding the selection to several neighboring residues to allow for contiguous stretches of residues and exclude single residue stretches, we used the following specifications for binding sites (in PDB numbering, from 1-N):

Antibody site:

45-62 EWMGWIKPERGAVSYAPQ

71-74 RDLY

101-105 RDASW

Antigen site:

160-167 NITNNAKI

222-228 SGGDLEI

276-281 NMWQRA

306-313 TRDGGKDN

324-327 GDMR

**Generating input files**

Next, we used a python script to generate Rosetta residue files (resfiles) for each virus that will be modeled. This script uses the provided multiple sequence alignment of the viral panel, titled 180_viruses_plus_4j6r.aln, to make a resfile to create each virus. The usage is shown below:

python2.7 make_viruses.py

This will create a directory called virus_resfiles, and within it a set of 180 resfiles. It will also create a file called viral_variants.fasta, with the concatenated binding sites of all viruses in the set.

Next, we used a similar script to make randomly mutated antibodies. The default setting is to make 5 random substitutions and 500 total antibody variants, but these can be specified in the script:

python2.7 make_antibody_variants.py 5 500

**Generating a training set with Rosetta**

At this point the files are prepared for Rosetta modeling of the complexes to generate a training set for SVM training and integer linear programming optimization. The input Rosetta XML file, options file, and command are provided in the protocol capture folder. The following command will generate 50 models for one antibody-antigen pair:

/path_to_rosetta/Rosetta/main/source/bin/rosetta_scripts.default.linuxgccrelease @relax_training.options -s 4j6r_renum.pdb -parser:protocol relax_training.xml -out:suffix _$abres"_"$virres -parser:script_vars abres=$abres -parser:script_vars virres=$virres

where abres has been set to an antibody variant resfile (e.g. ab-001) and virres has been set to a virus resfile (e.g. CAAN-A2).

**Training the Model**

Training involves a) classification to predict binding and b) regression to predict stability  scores. To perform training, we attach the following data: the set of the 30 virus sequences is in the file v_30.txt, the training set antibody and virus pairs and the corresponding scores are in train_set_ab_30.txt, train_set_v_30.txt, train_set_scores_binding_30.txt and train_set_scores_stability_30.txt respectively.

The following program creates feature sets from the training antibody and virus files for a given training size and then trains the machine learning models to predict binding and stability.

python train_models.py trainsize

The program outputs the coefficient and intercept terms for each machine learning model (classification and regression) and saves them to file. The variable ‘trainsize’ denotes the number of virus sequences chosen for training (on the corresponding antibody-virus pairwise datapoints). To execute the above program, scikit-learn needs to be installed on the system.

The following command learns prediction models on all the data:

python train_models.py 30

The above program learns a linear model in the default setting. To learn a non-linear model, the function scikit_programs_classification can be modified to choose the rbf kernel parameter. The parameters are set to the values mentioned in the paper. However, a 10-fold cross validation can be performed to optimize the parameter settings for a specific dataset.

The subset of 20 sequences is in v_20.txt. The training set antibody and virus pairs and the corresponding scores are in train_set_ab_20.txt, train_set_v_20.txt, train_set_scores_binding_20.txt and train_set_scores_stability_20.txt respectively. The following command learns prediction models on the subset of the data:

python train_models.py 20

The features used for training are saved in classification_features_trainsize.txt and regression_features_trainsize.txt. The parameters are saved in the format coefficients_trainsize_model.txt and intercept_trainsize_model.txt. Model maps to 1 for classification and 2 for regression.

**Integer Linear Program**

The following program reads the saved coefficients and intercepts, writes the integer linear program using cplex, solves it and writes the optimized antibody sequence to file. It needs IBM cplex to be installed on the system.

python solve_ILP.py trainsize

This writes the optimized antibody in BM_trainsize.txt (breadth maximized).

**Evaluating Optimized Antibodies**

Finally, the optimized antibodies can be evaluated using the saved model trained on all data. The following program computes the predicted breadth and the predicted stability score of the optimized antibody, and writes these numbers to file as predicted_breadth.txt and predicted_stability.txt.

python evaluate_optimized_antibody.py trainsize

**Running RECON multistate design**

To run RECON multistate design, you must first create an antibody-antigen complex for all the viral variants in the panel, using the crystal structure of VRC23 in PDB ID 4j6r as a template. Before running multistate design, we first threaded over the sequence of each viral variant and refined the complexes using a relax protocol with constraints to the starting backbone coordinates, to prevent too much movement of the protein backbone. We generated one relaxed model for each co-complex to use for design. Using the resfiles generated earlier, we use the following command to create the co-complexes:

/path_to_rosetta/Rosetta/main/source/bin/rosetta_scripts.default.linuxgccrelease @make_templates.options -s 4j6r_renum.pdb -parser:protocol make_templates.xml -out:suffix _$virres -parser:script_vars virres=$virres

where virres has been set to a virus resfile (e.g. CAAN-A2).

After generating the refined co-complexes we then performed RECON multistate design. The following command was used to run multistate design:

mpiexec -n 180 /path_to_rosetta/Rosetta/main/source/bin/rosetta_scripts.mpi.linuxgccrelease @msd.options -parser:protocol msd_brub.xml

The multistate design was run on a computing cluster with 180 processors allocated to the job. The residues allowed to design were the same residues in the paratope binding site as described previously, and the residues in the epitope binding site were allowed to repack. Backrub movements of the residues in the binding site of both the antibody and virus were performed in between rounds of design.

After generating multistate design models the top ten designs by overall fitness were selected and used to move forward to re-modeling in Rosetta to enable a direct comparison with the BROAD generated sequences. Overall fitness is defined as the sum of the Rosetta energy of all complexes included in multistate design. The following command was used to identify the top ten models in overall fitness, and to make a sequence logo for the top ten models:

grep CAAN msd.fasc | sort -nk19 | awk '{print $NF.pdb.gz}' | head -10 > msd_top10.list

python2.7 design_analysis.py --prefix msd_top10.list --res 4j6r.resfile --native 4j6r_renum.pdb `cat msd_top10.list`

**Evaluating breadth from Rosetta models**

After determining the best ten sequences predicted by both BROAD and multistate design, we subjected them to a more thorough modeling protocol to see if the sequences retain the predicted increases in breadth.

/path_to_rosetta/Rosetta/main/source/bin/rosetta_scripts.default.linuxgccrelease @relax_test.options -s 4j6r_renum.pdb –parser:protocol relax_test.xml -out:suffix _$abres"_"$virres -parser:script_vars abres=$abres -parser:script_vars virres=$virres

We next calculated the predicted breadth over the entire viral panel to determine which had greater predicted breadth. To do this we used the output of the testing set relaxation and measured the binding energy and score of the lowest scoring model for each antibody-antigen pair. We used the following script to do this analysis:

python2.7 compile_results.py relax_test.fasc

This script will calculate breadth over the whole panel for the native antibody and designed antibodies, and will output scatter plots showing the binding energy for each gp120 viral variant, before and after design, to see if design improved binding energy for this viral protein. A similar plot is also output showing the change in score instead of binding energy.
